# The Role of the Gut Microbiome in Youth with Polycystic Ovary Syndrome: A Systematic Review

**DOI:** 10.3390/children10121872

**Published:** 2023-11-29

**Authors:** Vasiliki-Rengina Tsinopoulou, Eleni P. Kotanidou, Nikolaos Athanasiadis, Evdoxia Sapountzi, Flora Bacopoulou, Evangelia Ntzani, Assimina Galli-Tsinopoulou, Athanasios Christoforidis

**Affiliations:** 1 Program of Postgraduate Studies “Adolescent Medicine and Adolescent Health Care”, School of Medicine, Faculty of Health Sciences, Aristotle University of Thessaloniki, 54124 Thessaloniki, Greece; epkotanidou@auth.gr (E.P.K.); agalli@auth.gr (A.G.-T.); christoforidis@auth.gr (A.C.); 2 2nd Department of Pediatrics, School of Medicine, Faculty of Health Sciences, Aristotle University of Thessaloniki, AHEPA University General Hospital, Stilponos Kyriakidi 1, 54636 Thessaloniki, Greece; 3 3rd Department of Obstetrics and Gynecology, School of Medicine, Faculty of Health Sciences, Aristotle University of Thessaloniki, Ippokration General Hospital, 54642 Thessaloniki, Greece; 4 Center for Adolescent Medicine and UNESCO Chair in Adolescent Health Care, 1st Department of Pediatrics, School of Medicine, National and Kapodistrian University of Athens, Aghia Sophia Children’s Hospital Athens, 11527 Athens, Greece; fbacopoulou@med.uoa.gr; 5 Department of Hygiene and Epidemiology, School of Medicine, University of Ioannina, 45500 Ioannina, Greece; entzani@uoi.gr; 6 1st Department of Pediatrics, School of Medicine, Faculty of Health Sciences, Aristotle University of Thessaloniki, Ippokration General Hospital, 54636 Thessaloniki, Greece

**Keywords:** adolescents, polycystic ovary syndrome (PCOS), microbiome, gut, dysbiosis

## Abstract

Background: Polycystic ovary syndrome (PCOS) is a common endocrine disorder that affects women of reproductive age and female adolescents. The diagnosis of PCOS is difficult during puberty due to overlapping of the criteria with normal variations of menstruation during this age period. There are insufficient data on the gut microbiome and PCOS and potential mechanisms linking the two. The present systematic review aimed to detect dysbiosis patterns in youth with PCOS, compared with healthy controls. Methods: One hundred seventy-eight studies were identified by a databases search and sixty-eight by a full-text assessment for eligibility; four were included in the systematic review and underwent quality control. Results: The results of the study were controversial in accordance to findings from the literature. A change in gut microbiome α diversity was found in PCOS adolescents, with no significant alterations in β diversity. Almost all studies found Firmicutes, Bacteroidetes, and Actinobacteria in abundance in both groups, with changes in family composition and fluctuations at the phylum level. A statistically significant association between these changes and clinical or biochemical features of the syndrome was described. Conclusions: This systematic review confirmed gut microbiota dysbiosis in youth with PCOS. However, further data are needed to clarify these changes and to build a strategy to prevent the syndrome.

## 1. Introduction

Polycystic ovary syndrome (PCOS) is a common endocrine disorder affecting women of reproductive age. The syndrome is usually established during adolescence and especially 2 years after the first menstruation. Data from around the world report that the disease prevalence varies between 6% and 9% of the population [1]. Accordingly, PCOS appears to be a popular diagnosis among adolescent females with a prevalence ranging from 3.4% to 11% depending on the diagnostic criteria used to establish the diagnosis [2].

Diagnostic criteria for PCOS include biochemical and/or clinical androgen excess, ovarian dysfunction, and ultrasonographic assessment of the polycystic ovarian morphology. Based on these criteria, known as the Rotterdam criteria, used to confirm the diagnosis of a female with PCOS, at least two of the aforementioned three criteria should be present [3].

In youth, the use of these diagnostic criteria is questionable due to the common presence of irregular and anovulatory menstrual cycles, acne—as a sign of hyperandrogenism—and pleiocystic ovarian morphology at this age [4]. Thus, the latest consensus on the diagnosis of PCOS during adolescence suggested to evaluate the coexistence of ovarian dysfunction, expressed as menstrual disturbances/oligomenorrhea, and biochemical hyperandrogenism in order to confirm the diagnosis of PCOS in youth [4,5].

Common ovarian pathologies in childhood and youth are reported as usually asymptomatic and they emerge only when complications occur, such as acute abdomen or a palpable mass in the ovarian lodge [6]. Unlike these situations, PCOS manifestation and complications include a broad group of manifestations, affecting both fertility and the metabolic profile. These complications change during the lifespan, beginning as menstrual irregularities, hirsutism, and infertility and then continuing as metabolic complications (glucose intolerance, type 2 diabetes), cardiac complications, and an increased incidence of endometrial cancer [7,8].

The quote “all diseases begin in the gut”, attributed to the Ancient Greek physician Hippocrates approximately 2500 years ago, seems to fit perfectly in the case of the role of the human gut in the pathogenesis of PCOS. Four dominant phyla of bacteria appear to colonize the human gut. The Firmicutes (Gram-positive, anaerobic/aerobic, saprophytic spore-forming bacteria, mainly represented by the genera Clostridium, Faecalibacterium, Blautia, Ruminococcus, Enterococcus, and Lactobacillus) and Bacteroidetes (Gram-negative, aerobic or anaerobic, non-spore-forming bacteria, represented by Bacteroides and Prevotella) [9,10] constitute approximately 90% of the normal bacterial flora of the small and large intestine [11]. The remaining two phyla that colonize the gastrointestinal tract are the Actinobacteria (Gram-positive bacteria, with the species of Bifidobacterium being the dominant species in the microflora of the newborn up to the first 1000 days of life) [12] and the Proteobacteria (Gram-negative bacteria, which show heterogeneity in morphology and physiological characteristics and consist of six different subclasses) [9].

However, the human gut microbiota is a growing and evolving ecosystem shaped by several factors during the lifespan, including the aging process, dietary habits, perinatal factors, sexual dimorphism, and hormonal factors [13]. Most diseases related to the gut microbiome result from either gut inflammation or dysbiosis [14].

It has been known for over two decades that the human gut microbiome plays a key role in the pathogenesis of PCOS—known as the DOGMA hypothesis (dysbiosis of the gut microbiota) [15]. Possible mechanisms include reduced intestinal mucosal permeability in obese populations or those on diets low in sugar, lipids, or dietary fibers and increased circulating lipopolysaccharides and, thus, insulin resistance and ovarian dysfunction [16]. Consequently, increased insulin levels lead to increased testosterone levels [17].

Few studies have so far focused on gut microbiome dysbiosis among youth with PCOS. Furthermore, different measures are often used to describe microbiome samples among studies, preventing the evaluation of available evidence on gut microbiota changes during PCOS. These measures may not provide information on the abundance of specific taxa, but reflect a change or difference in the composition of microorganisms. Gut microbiota diversity corresponds to the number of different species present in an individual. Alpha and beta diversity are the most specific indicators, describing the explored status of gut microbiota among different populations. The estimate of diversity in a single sample is called alpha diversity. Beta diversity analysis quantifies the similarity or distance between microbiome pairs [18] or between different samples [18]. Thus, alpha diversity measures the diversity of a particular population within the sample, while beta diversity measures are estimates of similarity between samples [18,19]. A systematic review in adult women with PCOS has demonstrated a significant alteration of the gut microbiota [20].

The aim of this study is to systematically review the available data on gut microbiome dysbiosis in female young people with PCOS.

## 2. Materials and Methods

The current systematic review was designed following a predefined protocol, according to the Preferred Reporting Items for Systematic Reviews and Meta-Analysis (PRISMA) guidelines, which is registered in the PROSPERO database under the identification number: CRD42023337850.

### 2.1. Search Strategy and Information Sources

The search for studies was based on a predefined P.I.C.O. (Population, Intervention, Comparison, Outcome) model. Based on this, the search included articles comparing young people (population) with PCOS (intervention) and those without (comparison), in which changes in gut microbiome composition (outcome) were investigated. Studies were searched in the scientific platforms PubMed, MEDLINE, and Cochrane Library, and all papers published between January 2012 and July 2023 were included. The search was limited to studies published in the English language. Specific keywords were used in the search filters: (“microbiome” OR “microbiota”) AND (“PCOS” OR “polycystic ovary syndrome”). Relevant articles from the reference sections of the screened articles that could be consistent with the subject of the systematic review were also included. In the case where more than one article was published from the same study, it was considered appropriate to extract data from the most recent and complete article.

### 2.2. Study Population Rationale and Eligibility Criteria

Studies considered eligible were randomized controlled trials (RCTs), cross-sectional studies, and cohort studies. According to the World Health Organization (WHO), the United Nations Population Fund (UNFPA), and the United Nations International Children’s Emergency Fund (UNICEF), young people are defined as a people between the ages of 10 and 24 years [21,22]. Thus, eligible studies included the age range of female patients with polycystic ovary syndrome up to 24 years.

Exclusion criteria related to study design, type of participants, and type of outcome were defined for the systematic review. Reviews (systematic or narrative), case reports or case series, case control studies, lead articles/opinion articles/commentary articles (editorial/commentary), and letters to the editor were excluded. In addition, studies involving an adult population, such as the age group over 24 years, were excluded. The maximum age of 24 years in the PCOS patient group was selected as a criterion in cases where the study design involved a youth group (referred to as either adolescents, young people, or youth). Studies performed in animals (mammals or rodents) were also excluded. Finally, studies where the end result was microbiome changes in systems other than the gut, such as changes in oropharyngeal or gynecological microbiome composition, were excluded from the present review.

### 2.3. Study Outcomes

The primary outcome was any reported change in the intestinal microbiome of patients with PCOS compared to controls. Secondary set outcomes were anthropometric factors and the hormonal and metabolic profile of the PCOS patients compared to controls.

### 2.4. Screening and Data Collection

Two authors with expertise in systematic reviewing screened all titles and abstracts for eligibility in a completely independent manner. Full texts were reviewed by the two reviewers and discrepancies were resolved with the involvement of a third reviewer. Reasons for exclusion were recorded for all studies excluded at the title, abstract, or full-text level of the review process. Data were extracted from full texts of studies into a predefined worksheet. Data collection was performed by two reviewers independently and then verified by a third according to the predefined datasheet. Any disagreements were resolved by discussion with the third investigator.

### 2.5. Quality Assessment of Included Studies

The risk of bias of the included studies was assessed using the ROBINS-I tool (Version 1 August 2016) (Risk of Bias in Nonrandomized Studies for Interventions) and the Cochrane RoB 2 tool for randomized trials [23,24]. ROBINS-I assesses seven different domains and scores studies as low, moderate, severe, and critical risk of bias [23]. According to the RoB2 tool, five domains are assessed and each domain is scored as low risk of bias, some concern, high risk of bias, or no information [24].

## 3. Results

Initially, the systematic search yielded 200 studies. Then, after excluding 51 duplicate studies, the abstracts of all remaining studies were checked. Those who met the entry criteria remained in the study and their entire text was thoroughly read, while those who did not meet the above entry criteria were excluded from further analysis. Of the rest, 75 were rejected because of a title unrelated to the search topic, but also after reading the abstract. The total number of papers studied at the full-text level was 74, of which 69 were rejected. Reasons for rejection included different type of study design (39), adult population studies (26), rat studies (1), looking for different results from the study outcome (2), but also having data based on a previous study included in the procedure (1). From the set of works that emerged, one was excluded; although it studied the population and the election result, it was in an indirect way.

More specifically, in the study by Eyupoglu et al. [25], potential changes in the intestinal microbiome composition of female adolescents with PCOS were studied by measuring trimethylamine N-oxide (TMAO), a gut microbiome-dependent metabolite [18]. In the study, the authors wanted to assess changes in the gut microbiome, expressed by variations in serum TMAO levels and its precursors [25].

**Figure 1:**
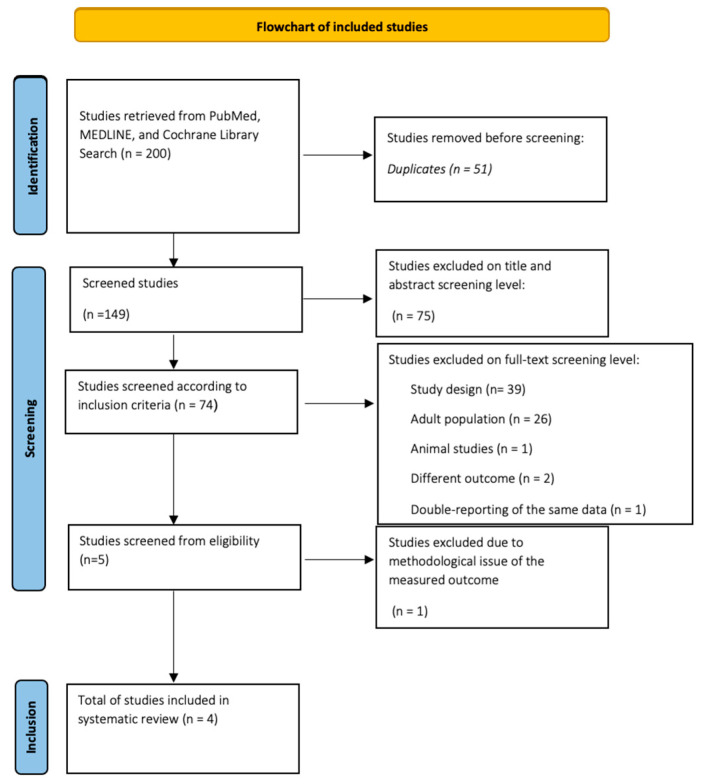
Flowchart of studies from databases.

Thus, four papers were finally included in the systematic review. The detailed PRISMA-compliant flow chart for inclusion and exclusion of potential published papers is shown in Figure 1.

A summary of the main characteristics of the studies included in the systematic review is presented in Table 1, Table 2, Table 3 and Table 4. Of the four total studies, two involved female youth with PCOs from Turkey [26,27], one from a similar population from Spain [28], and another with female adolescents from the United States of America [29].

In two out of four of the above studies, the diagnosis of PCOS was made based on the Rotterdam criteria [1,2]. In the study by Jobira et al. [29], the National Institute of Health (NIH) criteria were used, which were adapted to female adolescents with oligomenorrhea—at least 2 years after menarche and the presence of clinical and/or biochemical signs of hyperandrogenism. Garcia-Beltran et al. [28] used as inclusion criteria the presence of hirsutism, oligomenorrhoea, and at least 2 years since menarche [4]. Regarding the design of the studies, all were cross-sectional studies except for the study by Garcia-Beltran et al., which was a randomized controlled clinical trial [28].

Regarding the control groups, one study included adolescents of a similar age to the control group [28], another study involved adolescents with a similar BMI to the control group [27], while in another, the control group had a similar age and BMI to the study group [26]. The latter study reports participation in the control group of subjects from previous studies with recorded age, BMI, full puberty, and sedentary lifestyle not matched to the study group [29].

The age of patients with PCOS ranged from 15.5 to 22.5 years, while the age of controls ranged from 14.1 to 27 years. In Table 2, demographic and anthropometric characteristics of the included studies are presented, according to the age category of participants: adolescents or young people only. Finally, the BMI of the young participants with PCOS ranged from 19.7 to 39.7 kg/m^2^, while that of the control group ranged from 20 to 39.3 kg/m^2^.

In the randomized study by Garcia-Beltran et al. [28], gut microbiota dysbiosis was studied in female adolescents with PCOS, aiming for individualized therapeutic intervention.

The authors describe changes in α diversity at the level of uniformity and diversity (*p*= 0.03 and *p* = 0.04, respectively), while at the level of β diversity, they also show changes between the groups. Regarding the species of bacteria at the genus level, changes were observed in the genera of the XI family of Firmicutes (*p* = 0.002), where they were found in abundance in the group of adolescents with PCOS, in contrast to the control group. The genera Prevotellaceae of Bacteroidetes (*p* = 0.0006), Prevotella of Firmicutes (*p* = 0.0001), and Senegalimassilia of Actinobacteria (*p* = < 0.0001) were found in abundance.

Changes in α diversity, measured by Pielou’s evenness index, were correlated with free androgen index (FAI) (r = −0.288, *p* = 0.03) and SHBG levels (r = 0.323, *p* = 0.012), but also with alanine transaminase levels (ALT-r = 0.376 *p* = 0.003) [22]. Alterations of the genera Prevotellaceae and Prevotella were positively associated with SHBG levels (r = 0.301, *p* = 0.018 and r = 0.289, *p* = 0.02, respectively), LDL cholesterol levels (r = 0.297, *p* = 0.02 and r = 0.269, *p* = 0.036, respectively), and adiponectin levels (r = 0.322, *p* = 0.019 and r = 0.342, *p* = 0.013, respectively). Finally, changes in the genus Senegalimassilia were correlated with markers of hyperandrogenism such as testosterone levels (r = −0.284, *p* = 0.026), SHBG levels (r = 0.429, *p* = 0.001), FAI (r = −0.440, *p* = 0.001), liver fat (r = −0.352, *p* = 0.007), and central (visceral) fat (r = −0.309, *p* = 0.018). In the same study, the greatest difference in the gut microbiome composition between patients with PCOS and healthy controls was mostly determined by central fat distribution (F = 1.08, *p* = 0.008), liver fat (F = 1.32, *p* = 0.04), and free FAI (F = 1.05, *p* = 0.02) in a Bray–Curtis dissimilarity matrices analysis [28].

Similar findings of microbiome differentiation were also found in the study by Jobira et al. [29]. In this study, patients with PCOS showed reduced α diversity at the level of uniformity (*p* = 0.0052 and *p* = 0.045) compared to healthy individuals, without changes at the level of variety (*p* = 0.655).

The β diversity showed changes between the two groups (*p* < 0.001); regarding the sexes, adolescents with PCOS showed a greater abundance of Actinobacteria (*p* = 0.027), a lower presence of Bacteroidetes (*p* = 0.004), and similar levels of Firmicutes and Proteobacteria compared to the healthy subjects. At the family level, adolescents with PCOS had a lower abundance in Bacteroides (*p* < 0.001) and Porphyromonadaceae (*p* = 0.024), while opposite abundance in Streptococcaceae (*p* = 0.047). At the genus level, adolescents with PCOS had a higher abundance of Prevotella, Finegoldia, and Lactobacillus, but lower abundances of Bacteroides and Parabacteroides. In this study, levels of total testosterone, ALT, triglycerides (TG), and HOMA-IR were positively correlated with the changes. Additionally, changes that can potentially be used as predictors of PCOS were reported, such as the genera Bacteroidetes (AUC 0.73 ± 0.06) and Actinobacteria (AUC 0.68 ± 0.07), and the families Lactobacillaceae (AUC 0.75 ± 0.08), Bacteroidaceae (AUC 0.81 ± 0.06), Porphyromonadaceae (AUC 0.68 ± 0.07), and Streptococcaceae (AUC 0.66 ± 0.07), with the family Bacteroidaceae being the strongest predictor (sensitivity of 62% and a specificity of 86%).

However, the study by Eyupoglu et al. [23] presents opposite results, with α diversity showing no changes between the two compared groups (*p* = 0.27, *p* = 0.79, and *p* = 0.97). No change was showed in the β diversity either. At the genera level, there was also no differentiation between the groups, with Bacteroidetes and Firmicutes being abundant in both groups, but also with the presence of Proteobacteria and Actinobacteria. The only difference was found in the Ruminococcaceae family, where it was found in greater abundance in the PCOS group compared to the control group (*p* = 0.006). The abundance of this family was positively correlated with the score on the Ferriman–Gallwey scale (*p* = 0.01) [26].

The study by Mammadova et al. [27] in lean women with PCOS is consistent with the findings of the previous study, where there appears to be no difference between the two groups regarding α and β diversity (*p* = 0.78, *p* = 0.51, and *p* = 0.93, respectively). Regarding the sexes, patients with PCOS appear to have a greater abundance of Proteobacteria (*p* = 0.039), Gammaproteobacteria (*p* = 0.039), Erysipelotrichia (*p* = 0.013), and Verrucomicrobia (*p* = 0.05) compared to controls. In contrast, the genera Clostridium sensu stricto and Roseburia appear to be less abundant in the PCOs group compared to controls (*p* = 0.04 and *p* = 0.021, respectively) [27], as shown in Table 3.

Differences in α and β diversity are detailed in Table 4; however, it is important to underline that a pattern of significant differences is only reported among studies focusing on adolescence [28,29]. Interestingly, studies presenting data from young adults [26,27] failed to demonstrate any significant change between patients and controls, in either α diversity or β diversity.

Regarding study quality, three out of the four selected studies were assessed as low risk of bias by the ROBINS-I tool. The summary risk of the bias assessment using the ROBINS-I tool is reported in Table 5. One study was assessed as a randomized controlled trial by the RoB 2 tool, as shown in Table 6.

## 4. Discussion

In the present systematic review, an attempt was made to capture the existing research studies on intestinal microbiome variations in young female people with PCOS. The total number of women with PCOS that were included was 108, a relatively small sample, which also reflects the scarcity of studies on this specific topic. The small patient sample also reflects the difficulty of finding and conducting such studies in adolescent, non-adult populations. The difficulty is related both to the nature of the syndrome, where it is established in adolescents and young women after 2 years of menarche and therefore affects young women of older age in the majority, and to the lower prevalence of the syndrome in youth under 24 years of age.

In addition to the fact that PCOS is predominantly diagnosed in female youth rather than adolescents per se, the origin of the syndrome is clearly rooted in the metabolic profile of early adolescence [30]. This is why the manifestations of the syndrome preoccupy and trouble primarily the pediatrician dealing with the adolescent girl, rather than the adult medical provider who can easily establish the diagnosis. The rationale of the present study was to shed light on the etiological origin of the syndrome during adolescence through the pooling of the available evidence on gut dysbiosis in the context of PCOS. The data provided will mainly address the pediatric clinical perspective on the disease, reinforcing the underlying link of gut dysbiosis and the occurrence of PCOS. The aim of the researchers is to focus on the adolescent disorders associated with the syndrome in order to provide evidence for the design of effective interventions before the PCOS fully manifests during adult life [31]. Thus, the selection of the age range of the study population was based on the definitions of adolescents and youth according to global health promotion organizations and health stakeholders. According to the World Health Organization (WHO), the United Nations Population Fund (UNFPA), and the United Nations International Children’s Emergency Fund (UNICEF), young people are defined as people between the ages of 10 and 24 years. Thus, eligible studies included the age range of female patients with polycystic ovary syndrome up to 24 years.

The study design was initially based on setting the age of 24 as an entry criterion in order to include the population group that belongs to young adults (youth); in this way, the age spectrum of the onset of PCOS was extended to include changes in the microbiome, which are imminent to the aforementioned onset. However, even among the studies included here, a pattern of differences in findings was evident in relation to age variation. Studies in younger adolescents (under 17 years of age) tended to report significant differences in the diversity of the explored microbiota, while no significant alterations were found in more advanced age populations (young to 24 years of age) [26,27,28,29]. Age variation is a recognized factor that interferes with both phyla and their diversity in healthy conditions [32]. The entire age from infancy to the elderly is already known to be characterized by a different microbiome profile in healthy humans [32,33]. It can therefore be hypothesized that the effect of age variation on the microbiome may modify the investigated differences not only in health but also in disease.

It is well known that the study of the gut microbiome has been at the center of attention for some time, with data emerging highlighting changes initiated both by sexual dimorphism and by variations in the individual’s lifestyle and metabolic and endocrine profile, additionally modified after iatrogenic interventions [34].

Indeed, data from rodent studies support that the composition of the gut microbiome differs between sexes. The intestinal microbiome of the adult female mice appears similar to that of preadolescent mice, while adult male mice develop an intestinal environment distinct from that of preadolescent mice, regardless of sex [35,36].

Although studies on the effect of sexual dimorphism in humans are still scarce, according to the available data, the composition of the intestinal microbiome between women and men is reported to be diverse [37]. These differences may be due to the direct effect as well as the indirect influence of sex hormones on inflammatory and metabolic factors, such as short-chain fatty acids (SCFAs) and neurotransmitters. Furthermore, the diversity and composition of the microbiome, in addition to being related to age, also adapts to the effects of hormones. Mayneris-Perxachs et al. [38], in an attempt to investigate the changes in the composition of the gut microbiome between the two sexes (men and women), highlighted the differences in β diversity between premenopausal women and men, which is based on steroid biosynthesis, whereas on the contrary, no differences were observed between postmenopausal women and men.

These differences, however, were evident in non-obese subjects, disappeared in the obese population, and were strengthened by the positive correlation with sex steroids, progesterone, and testosterone levels. Gender differences were observed between premenopausal and postmenopausal women and men. Males showed greater abundance in Bacteroidaceae and Prevotellaceae, a finding reinforced by a possible positive covariance with testosterone. In contrast, the genera Actinobacteria, Proteobacteria, Firmicutes, and Verrucomicrobia were not associated with testosterone levels. Furthermore, estrogen levels differed in obese postmenopausal women compared to lean postmenopausal women; obese postmenopausal women have higher estrogen levels due to peripheral estrogen synthesis [38].

In the field of PCOS, however, many studies have attempted to highlight the changes occurring in the gut microbiome [39,40,41].

The particular difficulty in studying PCOS is related to its pathophysiology itself, where it is characterized by hyperandrogenemia, a factor that contributes to changing the composition of the intestinal microbiome but also to the frequent coexistence of insulin resistance and overweight or obesity (reported as up to 88% in adult PCOS) [42]. Recent meta-analysis provides evidence that there is a multiple relative risk of being diagnosed with either obesity, overweight, or central obesity in the setting of PCOS compared to healthy controls [43].

Studies in rodents have shown that changes in the gut microbiome, such as an increase in the genus Firmicutes, correlate with changes in the regulation of insulin levels, such as the presence or progression of obesity, type 2 diabetes, and the metabolic syndrome [44].

Zeng et al. reported changes in the functional and structural profile of the intestinal microbiome between women with PCOS and insulin resistance or without insulin resistance [39]. As a result, the presence of insulin resistance appears to directly affect the gut microbiome and act, possibly synergistically with PCOS, to further diversify the microbiota [45].

PCOS itself appears to enhance inflammation and insulin resistance due to a reduction in the abundance of beneficial bacteria for the microbiome (such as Faecalibacterium of the genus Firmicutes), thereby reducing the production of SCFAs that result in intestinal barrier disturbances [46].

Torres et al. conducted a study in healthy women, women with PCOS, and women with polycystic ovary morphology; they highlighted the link between hyperandrogenism and the changes that occur in the gut microbiome of women with PCOS. Differences occurred in four genes known to produce SCFAs, which were found at a lower rate in women with PCOS compared to the other examined groups [47].

Moreover, there is research on the presence of changes in the intestinal microflora of females with PCOS and overweight/obesity or lean weight. In a recent study by Liang et al. [48], gut microbiome changes were observed in Chinese women with PCOS and in healthy subjects and analyzed in relation to BMI levels.

In this study, the authors showed that the changes were present in both lean and obese women with PCOS. Especially, they showed statistically significant differences in bacterial relative abundance of the genera Bacteroidetes, Proteobacteria, and Parabacteroides in the entire sample of women with PCOS, regardless of BMI [43].

In addition, several research protocols are investigating the effect of contraceptive pills on the intestinal microflora. Hua et al. [49] reported that microbiome changes were observed before and after oral contraceptive administration. The results highlighted differences between the sexes and genera of the intestinal microbiome of women over time and the effect of contraception, particularly in the increase in the genera Actinobacteria and Firmicutes [49].

Another aspect of therapeutic personalization research is emerging with the primary goal of balancing microbiome diversity. The available data indicate that there is a tendency to direct therapeutic approaches targeting the relationship between microbiome and circulating androgens levels [50].

Regarding changes in the intestinal microbiome of female adolescents with PCOS, there is a great heterogeneity of the reported findings as a result of the limited number of relevant studies as well as the interpretation of physiological changes due to puberty.

One of these studies on adolescents with PCOS by Eyupoglu et al. [25] aimed to find changes in gut microbiome diversity by measuring serum levels of trimethylamine N-oxide (TMAO) for potential targeted therapy. TMAO is known to be produced from the metabolism of dietary choline and L-carnitine by the gut microbiome, and many studies have shown that this important product inhibits cholesterol metabolism, induces platelet aggregation and thrombosis, and promotes atherosclerosis. Moreover, TMAO levels, in addition to atherosclerosis, are associated with type 2 diabetes and gestational diabetes [51].

Studies in adult populations with PCOS have shown that TMAO levels are elevated, even without the clinical presence of hyperandrogenism [52].

The results of the study [25] were encouraging, as it was found that the elevated TMAO levels found in the group of adolescents with PCOS were reduced after short-term oral contraceptive therapy (3 months) combined with lifestyle changes. In addition, body weight loss and a decrease in circulating androgen levels were also positively correlated [25].

Another study also regarding adolescent females with PCOS reported that the coexistence of obesity and fatty liver infiltration may be related to the altered microbiome [53]. This study concluded that adolescents with PCOS and obesity and fatty liver infiltration have a different gut microbiota composition compared to those with PCOS and obesity [53].

The results obtained from the studies included in this review show great heterogeneity in terms of species diversity—findings that are in agreement with those reported in the literature. In most of these studies, when a change is described, it mainly focuses on the reduction in α diversity in women with PCOS [28,29].

Regarding β diversity and species diversity of the intestinal microbiome, studies by Garcia-Beltran et al. [28] and Eyupoglu et al. [26] were the ones that showed the most changes, especially in the genus Firmicutes, with differences in families and genera.

It is also important to point out that two of the studies included in the present systematic review studied the changes in the intestinal microbiome of female adolescents with PCOS and, in the process, sought to elucidate its changes after treatment.

The first study by Garcia-Beltran et al. [28] demonstrated gene-level changes, as mentioned above in family XI, which appear to be abundant in female adolescents with PCOS and decreased to return to normal levels after administration of a combination of drugs such as spironolactone, pioglitazone, and metformin for a time period of 1 year; however, when oral combined contraceptive therapy was administered, similar results were not observed. The significance of this finding is related to the role of family XI microbes in inflammatory liver diseases, hyperandrogenemia, and central fat distribution [28].

The second study by Eyupoglu et al. [26] studied the effect of oral contraceptives in female adolescents with PCOS. The results generally failed to show significant differences in the gut microbiome after 3 months of contraceptive treatment. Nevertheless, a sex-based trend for Actinobacteria has been reported in female young people with PCOS and obesity, accompanied by a decrease in body weight and androgen levels [26]. However, the authors point out the beneficial effect of oral contraceptive administration in reducing the abundance of the genus Actinobacteria based on the results of other studies [29,54,55], where an increase in the genus was observed, and not in their study.

## 5. Conclusions

Polycystic ovary syndrome is a common disease among young female people. The pathophysiological pathways leading to the clinical manifestations of the syndrome are still under investigation. Gut microflora dysbiosis has been explored as a major factor contributing to the pathogenesis of PCOS. Until now, studies focusing on gut microbiota changes in the young population with PCOS are few. The reduction in α diversity, but also changes in β diversity of the gut microbiome, in different families and genera, especially in the phylum Firmicutes, is confirmed in PCOS young people. Further data describing gut dysbiosis during PCOS in youth are of major importance, in order to build a strategy to prevent the syndrome.

## Figures and Tables

**Table 1:** Characteristics of the studies.

Author, Year	Country	Type of Study
Garcia-Beltran et al., 2020 [28]	Spain	Randomized controlled trial
Mammadova et al., 2020 [27]	Turkey	Cross-sectional
Jobira et al., 2020 [29]	U.S.A	Cross-sectional
Eyupoglu et al., 2020 [23]	Turkey	Cross-sectional

**Table 2:** Characteristics of the population involved in the study.

Author, Year	PCOS Group			Control Group		
	n	Age	BMI (kg/m^2^)	n	Age	BMI (kg/m^2^)
Adolescents						
Garcia-Beltran et al., 2020 [28]	30	15.8 (15.5–16.1)	25.0 ± 1.0	31	15.9 (15.7–16.1)	22.0 ± 0.0
Jobira et al., 2020 [29]	37	16.1 (15.8–16.4)	36.0 (32.9, 39.7)	21	14.5 (14.1–14.9)	35.0 (30.7, 39.3)
Young people						
Mammadova et al., 2020 [27]	24	19.5 (19–22.5)	22.9 ± 3.2	22	23.0 (22.0–24.3)	22.5 ± 2.5

Age is presented as median (min–max). BMI is presented as mean ± standard deviation or median (min–max) or median (25%, 75%), as appropriate.

**Table 3:** Synopsis of significant phyla differences between PCOS and control groups of included studies.

Author, Year	PCOS Phyla Differences
Adolescents	
Garcia-Beltran et al., 2020 [28]	↑ Family ΧΙ (Firmicutes) ↓ Prevotellaceae (Bacteroidetes) ↓ Prevotella (Firmicutes) ↓ Senegalimassilia (Actinobacteria)
Jobira et al., 2020 [29]	↑ Actinobacteria ↓ Bacteroidetes
Young people	
Mammadova et al., 2020 [27]	↑ Proteobacteria ↑ Gammaproteobacteria ↑ Erysipelotrichia ↑ Verrucomicrobia ↓ Roseburia ↓ Clostridium sensy stricto
Eyupoglu et al., 2020 [23]	↑ Ruminococcaceae (Firmicutes)

**Table 4:** Synopsis of diversity assessments in the included observational studies.

Author, Year	α Diversity	Β Diversity
Adolescents		
Garcia-Beltran et al., 2020 [28]	Significantly lower α diversity in PCOS patients compared to controls [Pielou’s Evenness Index (*p* = 0.03) and Shannon’s Index (*p* = 0.04)]	Significant differences in dispersion of dissimilarity matrices [Jaccard ANOSIM test (*p* = 0.001), Permanova test (*p* = 0.01), Permdisp test (*p* = 0.19), Bray–Curtis ANOSIM test (*p* = 0.001), Permanova test (*p* = 0.002), and Permdisp test (*p*= 0.0001)]
Jobira et al., 2020 [29]	Significantly lower α biodiversity in PCOS compared to controls [Evenness (*p* = 0.0052) and Shannon diversity (*p* = 0.045)]	Significant β diversity, reflecting overall gut microbial community composition [*p* < 0.001]
Young people		
Mammadova et al., 2020 [27]	No difference between PCOS patients and controls (*p* = 0.784)	Not significant difference between PCOS patients and controls (*p* = 0.937).
Eyupoglu et al., 2020 [23]	No difference between PCOS patients and controls [Faith PD (*p* = 0.27), Pielou evenness Index (*p*= 0.79), and Shannon Index (*p* = 0.97)]	Not significant difference between PCOS patients and controls [Bray–Curtis (*p* = 0.58), Jaccard index (*p* = 0.99), and UniFrac distances (*p* = 0.76)]

**Table 5:** Risk of bias assessment of included non-RCT studies.

	D1	D2	D3	D4	D5	D6	D7	Overall
Study								
Mammadova et al., 2020 [27]	🟢	🟢	🟢	🟡	🟢	🟢	🟡	🟢
Jobira et al., 2020 [29]	🟢	🟢	🟡	🟢	🟡	🟡	🟢	🟢
Eyupoglu et al., 2020 [23]	🟢	🟢	🟢	🟡	🟡	🟢	🟢	🟢
	Domains:							
	D1: Bias due to confounding		🟢 Low risk of bias					
	D2: Bias due to selection of participants		🟡 Moderate risk of bias					
	D3: Bias in classification of interventions		🔴 Serious risk of bias					
	D4: Bias due to deviations from intended interventions		⚫ Critical risk of bias					
	D5: Bias due to missing data		🔵 No information					
	D6: Bias in measurement of outcomes							
	D7: Bias in selection of the reported results							

**Table 6:** Risk of bias assessment of included RCT study.

	D1	D2	D3	D4	D5	Overall
Study						
Garcia-Beltran et al., 2020 [28]	🟡	🟢	🟢	🟡	🟢	🟢
	Domains:					
	D1: Bias due to randomization				🟢 Low	
	D2: Bias due to deviations from intended interventions				🟡 Some concerns	
	D3: Bias due to missing data				🔴 High	
	D4: Bias due to outcome measurement					
	D5: Bias in selection of the reported results					

## Data Availability

Data are unavailable due to privacy restrictions.
